# Training exposure, recovery, and injury incidence among course-based and competitive university Wushu Taolu practitioners: a 12-week prospective cohort study

**DOI:** 10.3389/fpubh.2026.1950026

**Published:** 2026-09-17

**Authors:** Qi Liu, Jiannan Yang

**Affiliations:** 1 Nanchang Institute of Technology, Jiangxi, China; 2 Hunan Normal University, Changsha, China

**Keywords:** musculoskeletal injury, prospective cohort, public health surveillance, recovery, training load, university athletes, Wushu Taolu

## Abstract

**Background:**

Whether the large training-exposure difference between course-based and competitive-team university Wushu Taolu practitioners in China translates into a correspondingly different musculoskeletal health burden has not been well characterized prospectively.

**Methods:**

In this observational, non-randomized cohort study, 255 practitioners (128 course-based, 127 university-team; mean age 20.7 ± 1.5) were followed over four waves (T0–T3) across 12 weeks, with training load, sleep, recovery, health problems, and injury recorded using recall windows matched to each variable’s time-scale.

**Results:**

University-team practitioners had substantially higher training exposure than course-based practitioners. The 12-week cumulative incidence of new injury was substantially higher in university-team practitioners than in course-based practitioners (68.9% vs. 20.0%; *p* < 0.001), with higher adjusted odds of injury (OR = 6.31, 95% CI 1.49–26.69). However, when injury occurrence was expressed relative to estimated training exposure, the between-group difference was much smaller: 10.56 versus 8.98 positive new-injury person-wave observations per 1,000 estimated training hours, respectively (IRR = 1.18, 95% CI 0.79–1.75). Prior injury within the preceding 12 months was strongly associated with subsequent injury (OR = 4.06, 95% CI 2.03–8.13).

**Conclusion:**

University-team practitioners experienced a substantially greater absolute injury burden than course-based practitioners, whereas the between-group difference was much smaller when injury occurrence was expressed relative to estimated training exposure. Prior injury history was a strong marker of subsequent injury. Because group membership and training exposure were highly correlated and the study was observational, these findings should not be interpreted causally.

## Introduction

Wushu Taolu combines high-amplitude jumps, rotations, and flexibility-dependent postures within short, high-intensity routines. University-level participation spans a wide exposure gradient: some students take Wushu as a general physical-education course with limited weekly contact time, while others are selected onto competitive university teams that train year-round at substantially higher volume and intensity. Whether these two exposure levels carry meaningfully different musculoskeletal health burdens is not a narrow sports-science question: Chinese universities differ widely in how medical coverage, injury-screening, and sports-medicine referral pathways are allocated between general physical-education courses and competitive teams, and if health burden differs substantially by training context, that has direct implications for how campus health services and injury-prevention resources should be prioritized.

From a public health perspective, injury and illness surveillance is considered a foundational element of protecting athlete health and informing preventive policy (1). Yet a recent systematic review of injury surveillance in university sport found that data collection practices remain inconsistent and poorly standardized across institutions, that most published surveillance systems originate from Western, team-sport contexts, and that only a small minority of studies conform to international consensus surveillance methodology (2). Individual, aesthetic-scoring, and culturally specific sports such as Wushu Taolu—and university settings outside North America and Europe more broadly—are correspondingly under-represented in this evidence base, despite Wushu’s status as a compulsory or elective component of physical education at many Chinese universities and its inclusion as a university-games event.

Existing evidence on Wushu-related musculoskeletal injury comes almost entirely from cross-sectional surveys conducted outside mainland China: studies of adult Wushu players in Iran and of youth competitive Wushu Taolu athletes in Singapore have reported musculoskeletal injury prevalence ranging from roughly 44 to 65%, depending on competitive level and country (3, 4), with the knee, ankle, and lower back consistently among the most commonly affected sites (4)—a pattern broadly consistent with the repetitive jumping, landing, and spinal-flexibility demands of taolu routines. Existing evidence on Wushu-related musculoskeletal injury has largely been derived from cross-sectional studies, leaving prospective comparisons between course-based and competitive-team university Wushu Taolu practitioners insufficiently characterized. The present study, therefore, addresses this gap by prospectively comparing these two training contexts within a single university using repeated, wave-matched assessments; the present study’s exposure comparison is, therefore, motivated as much by this documented gap in the international surveillance literature as by sport-specific injury patterns. Evidence from other combat and martial arts disciplines suggests that competitive status and greater training exposure are associated with a higher injury burden: a survey of mixed martial arts practitioners found significantly higher injury rates and a greater proportion of severe injuries among competitive athletes than among recreational practitioners (5), and a clinical review of youth martial arts participation similarly found that competitive, contact-based training carries substantially higher injury risk than non-competitive practice, with risk rising steeply once weekly training volume exceeds a threshold (6). Although these disciplines differ from Wushu Taolu—mixed martial arts is contact-based and the youth-focused review concerns a younger population—they support examining both training context and accumulated exposure rather than either alone. In the present study, group membership was treated as a contextual indicator encompassing training volume, technical difficulty, competitive demands, and selection, whereas exposure-adjusted injury rates were used to examine injury occurrence per unit of training time. These comparisons address complementary questions—which training context carries the greater absolute health burden, and whether injury risk per unit of training time differs—and were not interpreted as separable causal effects. Sleep has separately been identified as a modifiable correlate of injury risk in athletic populations, with shorter habitual sleep duration associated with greater injury likelihood in adolescent athletes (7), while other studies have reported associations between physical-activity intensity or participation and both sleep duration and sleep quality (8, 9). Together, these findings support considering sleep within the broader training–recovery context rather than solely as an injury-related factor. Accordingly, sleep duration was monitored longitudinally alongside training load and perceived recovery in the present study.

Monitoring training load and recovery alongside musculoskeletal health problems requires attention to recall windows: health-problem surveys administered only at widely spaced intervals risk missing injuries that resolve between assessments. The Oslo Sports Trauma Research Centre (OSTRC) approach was developed specifically to address this by combining a short (7-day) recall window with a severity-graded questionnaire capable of capturing both time-loss and non-time-loss health problems (10, 11). In the present study, an investigator-developed Chinese-language questionnaire informed by the conceptual structure of the OSTRC approach was used to capture both time-loss and non-time-loss health problems. Weekly training load was estimated using an adapted session-RPE-derived calculation based on training frequency, mean session duration, and a representative RPE rating for the preceding 7 days, drawing on the low-cost session-RPE approach to quantifying internal training load (12). The present study, therefore, used a four-wave design (T0–T3) over 12 weeks with recall periods matched to each variable’s natural time-scale (7 days for training, sleep, recovery, and current health problems; the inter-wave interval, approximately 4 weeks, for new injury onset and training days lost).

Conceptually, this study addresses the first two stages of the sequence-of-prevention framework: describing the extent and distribution of injury burden and exploring factors associated with that burden.

Building on this rationale, the aims of this study were to (i) describe and compare training load, sleep, and recovery trajectories between course-based and university-team Wushu Taolu practitioners over 12 weeks, with the expectation that the higher-exposure team group would show a consistently elevated load and reduced sleep/recovery profile; (ii) explore whether training load and recovery status measured at one wave were prospectively associated with study-defined substantial health problems at the subsequent wave; and (iii) identify baseline correlates—including group, prior injury, and participant and training-related characteristics—of experiencing at least one new injury over the 12-week follow-up, hypothesizing that university-team membership and a positive prior-injury history would show the strongest associations.

## Materials and methods

### Design and setting

This was a single-institution, observational, non-randomized prospective cohort study conducted over 12 weeks with four assessment waves (T0, T1, T2, and T3), reported in accordance with the STROBE guidelines for observational cohort studies (13). Group membership (physical-education course vs. university competitive team) was determined by participants’ existing enrollment status and was not assigned by the investigators; the study is, therefore, a naturalistic comparison of two existing exposure groups rather than a controlled intervention trial, consistent with the first two stages of the classical “sequence of prevention” model for sports injury research (14).

### Participants

The study was conducted at Hunan Normal University in Changsha, China. Baseline assessment (T0) was conducted on 2 May 2026, and follow-up assessments were conducted on 30 May 2026 (T1), 27 June 2026 (T2), and 25 July 2026 (T3), corresponding to approximately 4, 8, and 12 weeks after baseline.

Participants in the course-based group were recruited through announcements made during university Wushu Taolu physical education classes. Participants in the university-team group were recruited through the team coach from athletes listed on the official university-team roster. Eligibility criteria were: (1) being 18–23 years of age; (2) being a currently enrolled student at Hunan Normal University; (3) currently participating in a university Wushu Taolu physical-education course or training as a member of the university competitive team; and (4) voluntarily providing written informed consent. Participants were excluded if they were younger than 18 years, did not provide written informed consent, did not complete the baseline questionnaire, or if their membership in either the course-based or university-team group could not be verified.

Group membership was determined using official physical-education course-enrollment records for the course-based group and the official university-team roster, with confirmation by the team coach, for the competitive-team group. At each assessment wave, participants independently completed an electronic questionnaire. No researcher, course instructor, or coach was present while participants completed the questionnaire.

A total of 255 participants with valid baseline data were included in the analytic sample. One additional record in the longitudinal dataset was excluded because its participant ID had been entered incorrectly and could not be reliably linked to any baseline record during data cleaning. Consequently, the participant’s baseline characteristics, eligibility, and group membership could not be verified.

In total, 255 university Wushu Taolu practitioners aged 18–23 years (mean 20.7 ± 1.5) provided baseline data and were retained in the analytic sample: 128 enrolled in a general Wushu Taolu physical-education course and 127 members of the university competitive team; 128 were male and 127 female individuals. One additional record present in the longitudinal file could not be linked to a baseline record and was excluded prior to analysis, yielding a final analytic N of 255. As shown in Table 1, the two groups differed markedly at baseline: team members had approximately three times more taolu experience, were far more likely to have competition experience and to regularly perform high-difficulty movements, had a higher prevalence of injury in the preceding 12 months, and had slightly lower BMI than course-based participants (all *p* ≤ 0.009 except sex, which did not differ significantly). Group is, therefore, confounded with training history and experience from the outset, and this is treated as a central interpretive caveat throughout. Participant flow through enrollment, group allocation, and the four assessment waves is summarized in Figure 1.

**Table 1:** Baseline characteristics by group.

Characteristic	Course group (*n* = 128)	University team (*n* = 127)	*p*
Age, years, mean (SD)	20.8 (1.5)	20.6 (1.6)	0.282
Sex, male, n (%)	64 (50.0)	64 (50.4)	1.000
Height, cm, mean (SD)	168.6 (7.5)	169.2 (7.5)	0.478
Weight, kg, mean (SD)	63.3 (8.0)	61.8 (8.2)	0.154
BMI, kg/m^2^, mean (SD)	22.2 (2.2)	21.5 (2.1)	0.009
Taolu experience, months, mean (SD)	12.8 (6.3)	36.9 (15.7)	<0.001
Competition experience, n (%)	18 (14.1)	94 (74.0)	<0.001
Regularly performs high-difficulty movements, n (%)	34 (26.6)	93 (73.2)	<0.001
Injury in prior 12 months, n (%)	30 (23.4)	51 (40.2)	0.006

*p*-values from independent-samples *t*-tests (continuous) or chi-square tests (categorical).

**Figure 1:**
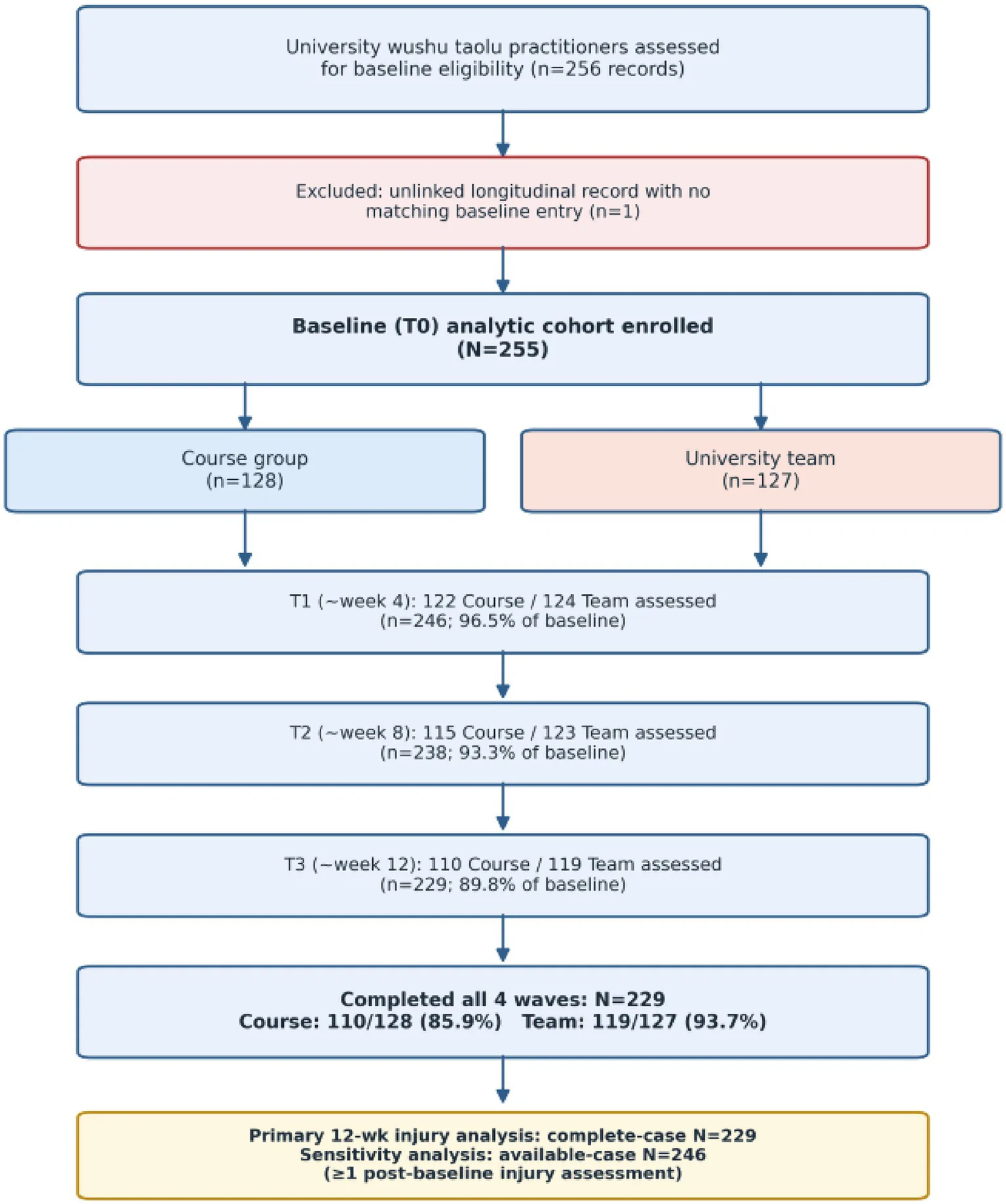
Participant flow through baseline enrollment, group allocation, and the four assessment waves (T0–T3).

### Assessment waves and recall windows

Assessments were conducted at baseline on 2 May 2026 (T0), followed by assessments on 30 May 2026 (T1), 27 June 2026 (T2), and 25 July 2026 (T3), corresponding to 4, 8, and 12 weeks after baseline, respectively. Retention was 255 (T0), 246 (T1), 238 (T2), and 229 (T3) completed assessments; 229 of 255 participants (89.8%) completed all four waves. To avoid under-counting injuries that occurred and resolved between waves, each follow-up wave included a dedicated “new injury/time-loss in the past 4 weeks” module in addition to the 7-day current-problem item. Recall windows were matched to each indicator’s natural time-scale: a 7-day window for training load, sleep, recovery, and current health problems, and a since-previous-wave window (approximately 4 weeks) for new injury onset and training days lost, so as to reduce recall bias and avoid missing injuries that resolved before a subsequent 7-day-recall assessment.

### Measures

Weekly training load was estimated using an adapted session-RPE-derived calculation based on training frequency, mean session duration, and a representative RPE rating for the preceding 7 days, drawing on the low-cost session-RPE approach to quantifying internal training load (12). This measure was derived from the session-RPE concept but was not equivalent to conventional session-RPE monitoring, in which session duration and RPE are recorded separately for each individual training session. Consequently, within-week variation in session duration and intensity was not captured, and the resulting value should be interpreted as an approximate weekly training-load indicator rather than a directly observed cumulative session-RPE load. Sleep was captured as mean nightly sleep duration (hours), derived from participants’ reported sleep over the preceding 7 days.

Health problems were assessed using an investigator-developed Chinese-language questionnaire informed by the conceptual structure of the Oslo Sports Trauma Research Centre (OSTRC) approach (10, 11), rather than the validated OSTRC questionnaire itself. The questionnaire was developed independently for the present study and was neither a direct translation nor an item-by-item adaptation of the validated OSTRC questionnaire. Four items assessed participation restriction, reduction in training volume, impaired performance, and symptoms during the preceding 7 days. The exact Chinese-language items and their English translations are provided in Supplementary material 1.

The four ordered response categories were scored 0, 8, 17, and 25, respectively, following the severity-grading structure of the OSTRC approach, and the four item scores were summed to produce a severity score ranging from 0 to 100. A substantial health problem was operationally defined as a moderate or severe reduction in training volume, a moderate or severe impairment in performance, or complete inability to participate in training or competition, corresponding to C2 ≥ 17, C3 ≥ 17, or C1 = 25. Because this was a study-developed, OSTRC-informed questionnaire rather than a formally validated Chinese version of the OSTRC instrument, its scores should not be regarded as psychometrically equivalent to those obtained using the validated OSTRC questionnaire. The questionnaire was developed in Chinese specifically for the present study. No formal pilot testing, structured expert content-validation procedure, forward–back translation, or separate psychometric validation study was conducted before data collection. The English wording provided in Supplementary material is a translation for reporting purposes and was not the version administered to participants.

Recovery, fatigue, and muscle soreness were each assessed as separate single-item 0–10 self-rated scales rather than as a multi-item composite. Recovery was assessed with a single item asking participants to rate their overall perceived recovery status over the preceding 7 days. Responses were recorded on an unanchored 0–10 numerical rating scale, with higher scores indicating better perceived recovery; no verbal endpoint anchors describing what 0 or 10 represented were provided to participants. Recovery was moderately-to-strongly correlated with fatigue (*r* = −0.71) and muscle soreness (*r* = −0.57), but was not perfectly collinear with either, consistent with these being related but distinct indicators. Fatigue and muscle soreness were treated as secondary descriptive indicators and were not modeled as primary outcomes or predictors.

New injury onset, injury site, injury type (acute vs. overuse), training days lost, and healthcare-seeking behavior were recorded for the interval since the previous wave (approximately 4 weeks), with injury and time-loss terminology aligned with the International Olympic Committee’s sport-generic consensus recommendations for injury and illness surveillance (1). A “new injury” was operationally defined as an injury that the participant reported as having occurred during that interval. The questionnaire did not separately ascertain whether a reported injury represented a recurrence or exacerbation of a previously reported injury, nor did it establish whether an injury reported at the preceding wave had fully resolved. Accordingly, repeated injury reports across different waves, including reports involving the same anatomical site, were treated as separate positive new-injury person-wave observations when participants reported a new injury in each interval; however, these observations cannot necessarily be assumed to represent clinically distinct injury events.

### Ethics statement

This study was reviewed and approved by the Institutional Ethics Committee of Hunan Normal University (approval no. 2026–363; approved 30 April 2026 and valid through 29 April 2027) and was conducted in accordance with the Declaration of Helsinki. Ethical approval was obtained before participant recruitment and data collection. Written informed consent was obtained from all participants before enrollment. Because all participants were 18–23 years of age, parental or guardian consent was not required. Baseline assessment was conducted on 2 May 2026, followed by assessments on 30 May 2026, 27 June 2026, and 25 July 2026. All assessments were conducted within the approved study period.

### Statistical analysis

All analyses were conducted in Python 3.11 using pandas 2.1 and SciPy 1.11 for descriptive and bivariate statistics and statsmodels 0.14 for regression, mixed-effects, and generalized estimating equation (GEE) models. Baseline group differences were tested with independent-samples t-tests for continuous variables and chi-square tests for categorical variables. Attrition was examined by comparing four-wave completers with non-completers on group, age, BMI, and prior injury; because the group difference in four-wave completion (85.9% course vs. 93.7% team) did not reach the conventional significance threshold (χ^2^ *p* = 0.066) but was numerically appreciable, residual attrition bias cannot be excluded and is treated as a limitation rather than ruled out. We additionally compared completers and non-completers on baseline (T0) substantial-health-problem status, and examined whether experiencing a new injury at the first follow-up wave (T1) was associated with subsequent non-completion of the remaining waves, as a further check for injury-related informative dropout.

For the repeated-measures models (mixed-effects and GEE, described below), missing waves were handled by maximum-likelihood/GEE estimation over all available observations for each participant (i.e., participants contributed data from whichever waves they completed); no imputation was performed. For the 12-week cumulative-injury outcome specifically, a participant’s injury status could not be established for waves they did not complete, so treating an unobserved wave as “no injury” would understate risk for those with partial follow-up. The complete-case analysis was selected as the primary analysis because it allowed cumulative injury status to be determined over the full 12-week follow-up for every participant included in that analysis. To assess the sensitivity of the findings to partial follow-up, a second analysis used the 246 participants with at least one post-baseline (T1–T3) injury assessment (available-case), treating unobserved waves as missing rather than injury-free; the remaining 9 of 255 participants had no post-baseline assessment and were necessarily excluded from both versions of this outcome. Because each wave’s new-injury item could register at most one “yes/no” outcome for that interval, a participant reporting one or more injuries within the same approximately 4-week interval contributed one positive new-injury person-wave observation. A participant reporting a new injury in more than one follow-up interval could therefore contribute more than one positive new-injury person-wave observation. The cumulative 12-week outcome was person-level (≥1 new injury during follow-up), whereas the injury-site/type summaries and exposure-adjusted rate analyses used the positive new-injury person-wave observations.

Repeated-measures outcomes (weekly training load, sleep hours, recovery score) were modeled with linear mixed-effects models with a random intercept for participant:

$$\begin{array}{l} Y\_\mathrm{it}=\beta 0+\beta 1\cdot \text{Time}+\beta 2\cdot \text{Group}\\ +\beta 3\cdot (\text{Time}\times \text{Group})+u\_i+\varepsilon \_\mathrm{it} \end{array}$$

Estimated by restricted maximum likelihood, with Time (T0–T3) and Group (course/team) as categorical fixed effects and their interaction; omnibus Time, Group, and Time × Group effects were evaluated with likelihood-ratio tests comparing nested models fit by maximum likelihood. Because the interaction model’s Group coefficient represents the group difference specifically at the T0 reference wave, we additionally fitted a main-effects-only (no-interaction) model to obtain a single additive estimate of the average group difference across all four waves, reported alongside the wave-specific contrasts.

Because the outcome of substantial health problems is binary and repeatedly measured, wave-level risk was modeled with a generalized estimating equation (GEE) logistic model with an exchangeable working correlation structure and robust (sandwich) standard errors, clustering on participant (15). Predictors were the previous wave’s training load (rescaled per 500 AU for interpretability) and recovery score (lag-1), prior 12-month injury, sex, group, and BMI; only waves T1–T3 (for which a lagged predictor was available) were included.

For the binary outcome of experiencing at least one new injury during the 12-week follow-up, a multivariable logistic regression was fitted with sex, group, taolu experience, baseline training load (per 500 AU), prior 12-month injury, baseline recovery, and BMI as predictors. Given 104 events among the primary complete-case sample of 229 participants (111 events among 246 in the available-case sensitivity sample) and seven predictors, this yields roughly 15 events per predictor, consistent with conventional guidance to limit model complexity relative to the number of events; it does not support extending the model with additional covariates or interaction terms. Because the cumulative injury outcome was common (occurring in over 40% of participants), odds ratios from this and the GEE health-problem model are interpreted throughout as measures of association and should not be interpreted directly as risk ratios; we therefore consistently use “odds,” “odds ratio,” and “associated with higher odds” rather than risk-ratio language (e.g., “risk increased X-fold”) when describing these results. To supplement the odds ratios for the complete-case cumulative-injury outcome, we additionally report the crude absolute risk difference and relative risk between groups (with Wald and log-transformed 95% confidence intervals, respectively), and a multivariable-adjusted absolute risk difference and relative risk obtained by standardization (g-computation): predicted probabilities from the fitted multivariable logistic model were computed for every participant under the counterfactual conditions of team and course membership, holding all other covariates at their observed values, and averaged; 95% confidence intervals for the adjusted estimates were obtained via 2,000 bootstrap resamples of participants.

Because university-team membership was associated with a roughly four-fold higher baseline training load (team vs. course ratio ≈3.9), we assessed collinearity between group and training load before interpreting their independent regression coefficients: the point-biserial correlation between group and baseline training load was *r* = 0.88, and the variance inflation factor for group and for baseline load in the multivariable injury model were approximately 5.5 and 5.0, respectively, indicating substantial shared variance and limited ability to statistically separate the associations of group membership and training load. Accordingly, coefficients from models containing both variables were interpreted as conditional associations between strongly overlapping predictors rather than as estimates of independent or causal contributions. Given this, in addition to the full multivariable models we fitted group-only, load-only, and group-plus-load models for both the cumulative-injury and wave-level health-problem outcomes to characterize how much each variable’s association depended on the other’s presence in the model; these comparisons are reported in Results 3.3–3.4 rather than assumed.

To separate exposure (how much a participant trained) from group membership as a label, we additionally modeled positive new-injury person-wave observations using a person-period Poisson model with log training hours as an offset and participant-clustered robust standard errors: each participant contributed one record per completed post-baseline wave (T1, T2, and/or T3), with that wave’s binary injury indicator as the outcome. Each record’s exposure was each participant’s weekly training hours at that wave (session count × mean session duration, converted to hours) multiplied by approximately 4 weeks to reconstruct that wave’s interval exposure; this is a coarse approximation because it assumes a constant weekly training pattern within each 4-week interval and does not capture week-to-week variation, and should be interpreted accordingly. The numerator for this analysis was therefore the total count of positive new-injury person-wave observations, which could exceed the number of participants with at least one injury if some participants reported a new injury in more than one wave. A Poisson model with log-exposure-hours as an offset and participant-clustered robust standard errors was fitted to obtain a group incidence rate ratio (IRR). As a sensitivity analysis, interval training exposure was alternatively reconstructed as four times the mean of the weekly training hours reported at the beginning and end of each approximately 4-week interval (e.g., the mean of the T0 and T1 values for the T0–T1 interval), allowing exposure to vary between adjacent assessment waves rather than assuming that a single end-of-interval observation represented the entire interval; the person-period Poisson model was then refitted using this alternative exposure estimate as the log-offset.

Two-sided *p* < 0.05 was considered statistically significant; given the exploratory, hypothesis-generating nature of the secondary models, results are reported with effect sizes and 95% confidence intervals throughout rather than relying on significance thresholds alone, and are not adjusted for multiple comparisons across the three modeling stages. Linearity in the logit was assessed for the continuous predictors in the primary cumulative-injury model (taolu experience, baseline training load, baseline recovery, and BMI) using the Box–Tidwell test, in which each predictor’s product with its natural logarithm was added to the model; a statistically significant interaction term would indicate departure from linearity. Because two participants had a baseline recovery score of 0, a constant of 1 was added to this variable before the log transformation for this test only. This check was not performed for the wave-level GEE health-problem model. All fitted models converged. For the linear mixed-effects models of sleep duration and recovery score, the random-intercept variance was estimated at or near zero, indicating limited residual between-participant heterogeneity after accounting for the fixed effects. As a sensitivity analysis, these models were refitted as a Gaussian generalized estimating equation with an exchangeable working correlation and as an ordinary least-squares model with participant-clustered robust standard errors; both approaches yielded fixed-effect estimates, confidence intervals, and *p*-values essentially identical to the mixed-effects results, indicating that the boundary variance estimate did not materially affect inference. Residual diagnostics (residual-versus-fitted plots and Q–Q plots) for both models showed no evidence of marked heteroscedasticity or non-normality. Multicollinearity was assessed using variance inflation factors as described above.

## Results

### Sample and retention

The analytic sample comprised 255 participants (128 course, 127 team; 64/64 male/female in the course group and 64/63 male/female in the university-team group—see Table 1). Retention across the four waves was high (255, 246, 238, 229 completed assessments at T0–T3, respectively), with 229 participants (89.8%) completing all four waves. Four-wave completion did not reach the conventional threshold for a statistically significant group difference (85.9% course vs. 93.7% team; χ^2^ *p* = 0.066); however, completion was numerically lower in the course group, and residual attrition bias cannot be excluded. Completion also did not differ significantly by age (*p* = 0.486), BMI (*p* = 0.785), or prior injury history (*p* = 1.00). Non-completers had a lower prevalence of a substantial health problem at baseline than completers (0/26, 0.0% vs. 37/229, 16.2%; χ^2^ *p* = 0.027). Among the 246 participants with a T1 assessment, those who reported a new injury at T1 subsequently dropped out of the study somewhat more often than those who did not (11.9% vs. 5.9%; χ^2^ *p* = 0.161). Baseline characteristics by group are shown in Table 1.

### Training load, sleep, and recovery trajectories

Averaged across all four waves, university-team members trained at markedly higher load than course-based participants (additive model average difference 2008.1 AU/week, 95% CI 1935.9–2080.3, *p* < 0.001; the group contrast specifically at T0 was 2026.2 AU/week), slept less (average difference −0.35 h/night, 95% CI−0.43 to −0.27, *p* < 0.001), and reported lower recovery (average difference −1.71 points on the 0–10 scale, 95% CI−1.88 to −1.55, *p* < 0.001). There were significant main effects of time on training load and sleep (both *p* < 0.001) and a borderline effect on recovery (*p* = 0.056), reflecting a period of elevated load and reduced sleep/recovery around T2 (week 8) in both groups. A significant Time × Group interaction was observed for training load (*p* = 0.011), indicating that the load trajectory diverged somewhat between groups over the 12 weeks, whereas the Time × Group interaction was not significant for sleep (*p* = 0.289) or recovery (*p* = 0.243), indicating broadly parallel trajectories despite the large between-group offset. Because group membership and training load were themselves highly correlated at baseline (point-biserial *r* = 0.88; see Wave-level health problem risk and Cumulative injury over 12 weeks, below), the group and time-varying-load estimates reported here describe two highly overlapping ways of characterizing the same underlying exposure difference rather than fully independent effects. Full model estimates, including all wave-specific contrasts and interaction terms, are reported in Tables 2–4 and depicted in Figure 2.

**Table 2:** Linear mixed-effects model for weekly training load (AU) (random intercept per participant; reference: T0, course group).

Parameter	Estimate	95% CI	*z*	*p*
T1 vs. T0	46.5	−81.3, 174.3	0.71	0.475
T2 vs. T0	170.5	40.6, 300.4	2.57	0.010
T3 vs. T0	−34.5	−166.0, 97.1	−0.51	0.608
Group, at T0	2026.2	1896.0, 2156.5	30.50	<0.001
T1 × group	−151.8	−332.3, 28.8	−1.65	0.099
T2 × group	151.2	−31.0, 333.4	1.62	0.104
T3 × group	−70.8	−254.9, 113.4	−0.75	0.451
Omnibus time (3 df, LR)	–	–	–	<0.001
Omnibus time × group (3 df, LR)	–	–	–	0.011
Avg. group effect (no-interaction model)	2008.1	1935.9, 2080.3	–	<0.001

**Table 3:** Linear mixed-effects model for sleep duration (h/night) (random intercept per participant; reference: T0, Course group).

Parameter	Estimate	95% CI	*z*	*p*
T1 vs. T0	0.02	−0.14, 0.18	0.22	0.828
T2 vs. T0	−0.18	−0.34, −0.02	−2.15	0.032
T3 vs. T0	0.12	−0.05, 0.28	1.42	0.157
Group, at T0	−0.27	−0.43, −0.11	−3.29	0.001
T1 × group	−0.04	−0.27, 0.19	−0.33	0.742
T2 × group	−0.10	−0.33, 0.13	−0.85	0.396
T3 × group	−0.21	−0.45, 0.02	−1.82	0.069
Omnibus time (3 df, LR)	–	–	–	<0.001
Omnibus time × group (3 df, LR)	–	–	–	0.289
Avg. group effect (no-interaction model)	−0.35	−0.43, −0.27	–	<0.001

**Table 4:** Linear mixed-effects model for recovery (0–10) (random intercept per participant; reference: T0, Course group).

Parameter	Estimate	95% CI	*z*	*p*
T1 vs. T0	0.19	−0.14, 0.51	1.13	0.257
T2 vs. T0	0.08	−0.25, 0.40	0.45	0.651
T3 vs. T0	0.39	0.05, 0.72	2.27	0.023
Group, at T0	−1.47	−1.79, −1.15	−9.01	<0.001
T1 × group	−0.19	−0.64, 0.27	−0.80	0.422
T2 × group	−0.41	−0.87, 0.05	−1.76	0.079
T3 × group	−0.40	−0.87, 0.07	−1.69	0.092
Omnibus time (3 df, LR)	–	–	–	0.056
Omnibus time × Group (3 df, LR)	–	–	–	0.243
Avg. group effect (no-interaction model)	−1.71	−1.88, −1.55	–	<0.001

Time and Time × Group omnibus *p*-values are from likelihood-ratio tests comparing nested models fit by maximum likelihood (3 df each); individual wave contrasts are Wald tests from the REML-fitted model. AU, arbitrary units.

**Figure 2:**
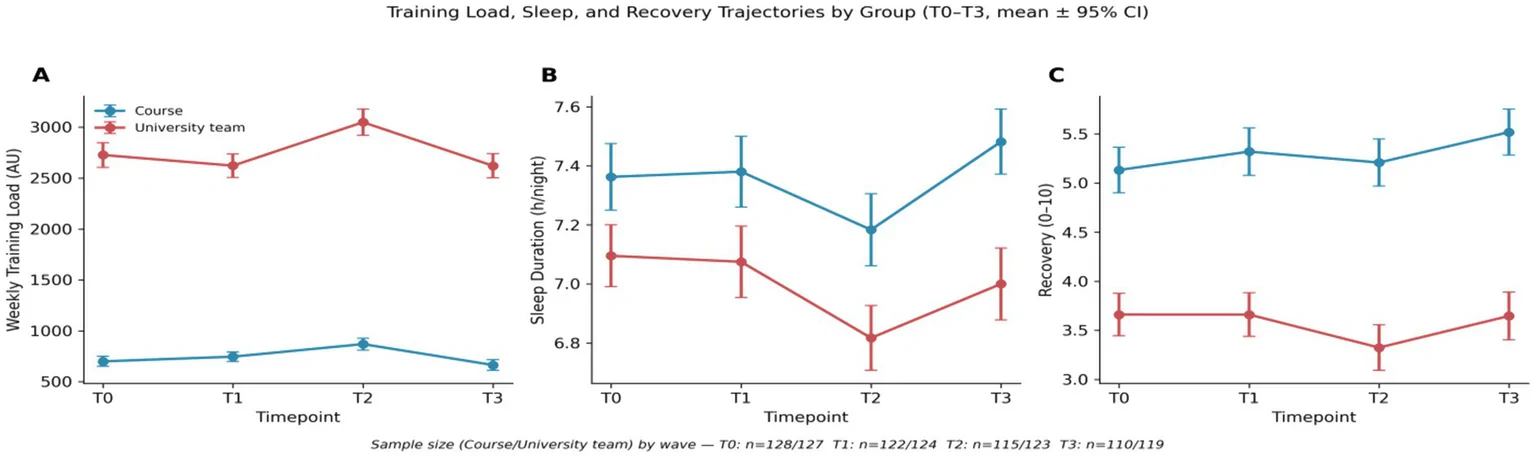
Mean (95% CI) values by group across the four assessment waves (T0–T3). **(A)** weekly training load, **(B)** sleep duration, and **(C)** recovery score.

### Wave-level health problem risk

Substantial health problems were reported by 2.6–7.8% of course-based participants across waves (T0 10/128 = 7.8%, T1 4/122 = 3.3%, T2 3/115 = 2.6%, T3 4/110 = 3.6%) and by 21.3–31.7% of team members (T0 27/127 = 21.3%, T1 28/124 = 22.6%, T2 39/123 = 31.7%, T3 35/119 = 29.4%); the team-to-course prevalence ratio ranged from roughly 2.7-fold at T0 to 12.2-fold at T2 across the four waves. In the lagged GEE logistic model (713 person-wave observations from 246 participants with a prior wave available), group membership was associated with substantial health problems at the following wave (OR = 10.47, 95% CI 4.26–25.76, *p* < 0.001). After group membership was included in the model, the association for the previous wave’s training load was substantially attenuated (per 500 AU: OR = 1.01, 95% CI 0.85–1.20, *p* = 0.895), and recovery score showed little association (OR = 0.99, *p* = 0.906); prior injury (OR = 1.49, *p* = 0.106), sex (OR = 0.87, *p* = 0.569), and BMI (OR = 1.01, *p* = 0.860) were also not statistically significant. Given the strong correlation between group membership and training load, these coefficients should be interpreted as conditional associations between overlapping predictors rather than as statistically separable independent effects. Full results are shown in Table 5.

**Table 5:** GEE logistic model (exchangeable working correlation) for substantial health problem at wave t from wave t − 1 predictors (waves T1–T3).

Predictor (wave t, lag 1 = wave t − 1)	OR	95% CI	*p*
Training load, wave t − 1 (per 500 AU)	1.01	0.85, 1.20	0.895
Recovery score, wave t − 1 (per point)	0.99	0.84, 1.17	0.906
Prior injury (past 12 months)	1.49	0.92, 2.41	0.106
Sex (male vs. female)	0.87	0.55, 1.39	0.569
Group (team vs. course)	10.47	4.26, 25.76	<0.001
BMI (per kg/m^2^)	1.01	0.91, 1.12	0.860

Because group and the previous wave’s training load were strongly correlated, we compared group-only, load-only, and group-plus-load GEE models (full estimates with 95% CI in Table 6) to assess how much each estimate depended on the other’s presence. Group alone showed OR = 11.77 (95% CI 6.25–22.18, *p* < 0.001); training load alone (per 500 AU) showed OR = 1.42 (95% CI 1.29–1.55, *p* < 0.001); with both in the model, the group estimate was little changed (OR = 11.22, 95% CI 4.63–27.15, *p* < 0.001) while the load estimate attenuated to near null (OR = 1.01, 95% CI 0.86–1.19, *p* = 0.884). This pattern indicates that training load’s univariate association with subsequent health problems overlaps very substantially with group, though—given the observational design and the absence of a formal mediation analysis—we do not interpret this as proof that load operates only through group.

**Table 6:** Group-only, load-only, and combined models for both wave-level health problems (GEE) and 12-week injury (complete-case, *N* = 229), illustrating group–load collinearity.

Model	Group OR (95% CI)	Load OR/500 AU (95% CI)	p (group/load)
Health problem (GEE), group only	11.77 (6.25, 22.18)	–	<0.001/–
Health problem (GEE), load only	–	1.42 (1.29, 1.55)	–/<0.001
Health problem (GEE), group + load	11.22 (4.63, 27.15)	1.01 (0.86, 1.19)	<0.001/0.884
12-wk injury (*N* = 229), group only	8.86 (4.83, 16.27)	–	<0.001/–
12-wk injury (*N* = 229), load only	–	1.54 (1.35, 1.77)	–/<0.001
12-wk injury (*N* = 229), group + load	6.98 (2.08, 23.44)	1.06 (0.82, 1.37)	0.002/0.657

### Cumulative injury over 12 weeks

Defining the 12-week injury outcome requires specifying the analytic sample precisely, because a participant who did not complete a wave has an unknown injury status for that interval rather than a confirmed absence of injury. We, therefore, report two versions of this analysis. The primary analysis is restricted to the 229 participants who completed all four waves (complete-case): 104/229 (45.4%) reported ≥1 new injury (course 22/110, 20.0% vs. team 82/119, 68.9%; χ^2^ *p* < 0.001; absolute risk difference 48.9 percentage points, 95% CI 37.7–60.1; relative risk 3.45, 95% CI 2.33–5.10). A sensitivity analysis includes the 246 participants with at least one post-baseline (T1–T3) injury assessment (available-case; this is the maximum possible denominator given that only 246/255 participants have any post-baseline data—see Figure 1): 111/246 (45.1%) reported ≥1 new injury (course 26/122, 21.3% vs. team 85/124, 68.5%; χ^2^ *p* < 0.001), for participants with a confirmed injury status on at least one occasion; participants with no post-baseline assessment at all (*n* = 9) are necessarily excluded from both versions rather than assumed injury-free. The closely consistent available-case results support the robustness of the primary complete-case findings to the alternative handling of participants with partial follow-up (see Tables 7, 8 for full results). Mean training days lost was correspondingly higher in the team group (6.7 ± 6.7 days, median 5) than the course group (1.2 ± 2.4 days, median 0; Mann–Whitney *p* < 0.001).

**Table 7:** Multivariable logistic regression for ≥1 new injury over 12 weeks—primary analysis (complete-case, *N* = 229, 104 events).

Predictor (baseline)	OR	95% CI	*p*
Sex (male vs. female)	1.15	0.61, 2.15	0.666
Group (team vs. course)	6.31	1.49, 26.69	0.012
Taolu experience (per month)	0.99	0.97, 1.02	0.658
Baseline training load (per 500 AU)	1.06	0.79, 1.42	0.709
Prior injury (past 12 months)	4.06	2.03, 8.13	<0.001
Baseline recovery (per point)	0.85	0.66, 1.11	0.234
BMI (per kg/m^2^)	1.04	0.90, 1.21	0.606

**Table 8:** Multivariable logistic regression for ≥1 new injury over 12 weeks—sensitivity analysis (available-case, *N* = 246, 111 events).

Predictor (baseline)	OR	95% CI	*P*
Sex (male vs. female)	0.98	0.54, 1.77	0.942
Group (team vs. course)	5.24	1.32, 20.73	0.018
Taolu experience (per month)	1.00	0.97, 1.02	0.718
Baseline training load (per 500 AU)	1.05	0.79, 1.40	0.724
Prior injury (past 12 months)	3.77	1.95, 7.29	<0.001
Baseline recovery (per point)	0.83	0.65, 1.06	0.131
BMI (per kg/m^2^)	1.01	0.88, 1.17	0.849

Among the 141 positive new-injury person-wave observations recorded (a participant reporting a new injury in more than one wave contributed more than one observation, whereas only one positive injury observation could be recorded per participant per wave), the most frequently affected sites were the lower back (*n* = 38), knee (*n* = 37), ankle (*n* = 28), wrist/hand (*n* = 13), hip/groin (*n* = 11), shoulder (*n* = 8), and other sites (*n* = 6); overuse-type injuries (*n* = 88) were more common than acute injuries (*n* = 53), and 106/141 observations (75.2%) involved measurable training time loss. Healthcare seeking was reported in 89 of 150 substantial-health-problem observations (59.3%); because the same participant could contribute more than one such observation across waves, this proportion describes observation-level rather than person-level healthcare-seeking behavior.

Because raw injury counts conflate how much a group trained with how risky that training was per unit of exposure, we modeled positive new-injury person-wave observations using a person-period Poisson model with log training hours as an offset and participant-clustered robust standard errors. Each participant contributed one record per completed post-baseline wave (713 person-period observations from 246 participants, matching the Wave-level health problem risk analysis above), with that wave’s injury indicator as the outcome and log reconstructed training hours for that approximately 4-week interval as the offset. This distinguishes the person-level outcome of “at least one injury” from the 141 positive new-injury person-wave observations used in the rate calculation (111 participants with ≥1 injury vs. 141 positive person-wave observations). Based on the reconstructed training exposure, the course group was estimated to have accumulated approximately 3,451 training hours over the observed follow-up, with 31 positive new-injury person-wave observations (8.98 per 1,000 estimated training hours), whereas the team group was estimated to have accumulated approximately 10,415 training hours, with 110 positive new-injury person-wave observations (10.56 per 1,000 estimated training hours). The resulting incidence rate ratio (team vs. course) was 1.18 (95% CI 0.79–1.75, *p* = 0.42). In the sensitivity analysis, in which each interval’s exposure was reconstructed as four times the mean of the weekly training hours measured at the beginning and end of that interval, the course group was estimated to have accumulated approximately 3,449 h with 31 positive new-injury person-wave observations (8.99 per 1,000 estimated training hours), and the team group approximately 10,442 h with 110 positive new-injury person-wave observations (10.54 per 1,000 estimated training hours), yielding an IRR of 1.17 (95% CI 0.79–1.74, *p* = 0.43). The magnitude and interpretation of the estimate were therefore materially unchanged. This confidence interval is wide and includes both a substantially lower and a substantially higher exposure-adjusted rate in the team group, so this analysis does not provide clear statistical evidence of a between-group difference in the per-hour injury rate, but it likewise does not establish that the rates are equal—a formal equivalence test with a pre-specified margin would be needed to support an “equivalent risk” conclusion, which we have not conducted. What this analysis does support is that the roughly three-fold gap in raw 12-week injury prevalence between groups is not similarly large when injury occurrence is expressed relative to estimated training exposure. Because the exposure values were reconstructed from 7-day training reports rather than directly recorded across each 4-week interval, the exposure-adjusted findings should be interpreted as indicative rather than definitive.

In the primary (complete-case, *N* = 229) multivariable logistic regression for any new injury over 12 weeks (104 events), group membership (OR = 6.31, 95% CI 1.49–26.69, *p* = 0.012) and prior 12-month injury (OR = 4.06, 95% CI 2.03–8.13, *p* < 0.001) remained associated with higher odds of injury. Because course and team groups differed substantially at baseline in training experience, competition history, high-difficulty movement exposure, and prior injury (Table 1), this odds ratio should not be read as indicating that group membership itself is the operative exposure driving injury risk, but rather that it summarizes a bundle of correlated between-group differences. Sex, taolu experience, baseline training load (per 500 AU: OR = 1.06, 95% CI 0.79–1.42, *p* = 0.709), baseline recovery, and BMI were not significant (all *p* > 0.23). Because the cumulative-injury outcome was common (occurring in over 40% of the complete-case sample), the odds ratio can overstate the corresponding risk ratio; using multivariable-adjusted standardization (g-computation from the fitted model, holding all other covariates at their observed values), the model-predicted risk of injury was 63.5% under team membership versus 25.1% under course membership, corresponding to an adjusted absolute risk difference of 38.4 percentage points (95% bootstrap CI 8.2–63.4) and an adjusted relative risk of 2.53 (95% bootstrap CI 1.19–5.89). The available-case sensitivity analysis (*N* = 246, 111 events; Table 8) showed a closely consistent pattern (group OR = 5.24, 95% CI 1.32–20.73, *p* = 0.018; prior injury OR = 3.77, 95% CI 1.95–7.29, *p* < 0.001). As in the Wave-level health problem risk analysis above, group and baseline training load were strongly correlated (point-biserial *r* = 0.88 at baseline; variance inflation factors ≈5.5 for group and ≈5.0 for load), so their coefficients should not be interpreted as fully independent effects. In group-only, load-only, and group-plus-load comparison models (Table 6), group alone showed OR = 8.86 (95% CI 4.83–16.27, *p* < 0.001), load alone (per 500 AU) showed OR = 1.54 (95% CI 1.35–1.77, *p* < 0.001), and with both included the group estimate remained significant though attenuated (OR = 6.98, 95% CI 2.08–23.44, *p* = 0.002) while the load estimate attenuated toward null (OR = 1.06, 95% CI 0.82–1.37, *p* = 0.657) — the same pattern observed in the health-problem model, and broadly consistent with, though not proof of, the exposure-adjusted rate finding above. A Box–Tidwell test found no statistically significant departure from linearity in the logit for the continuous predictors in this model (taolu experience, baseline training load, baseline recovery, and BMI; all interaction *p* ≥ 0.17), providing no evidence against the linearity assumption. Full primary results are shown in Table 7.

## Discussion

In this 12-week prospective cohort of 255 university Wushu Taolu practitioners, competitive university-team members showed a substantially higher unadjusted training and health-problem burden than course-based participants: roughly 3.5- to 4-fold the weekly training load, modestly less sleep, lower recovery scores, and several-fold higher rates of substantial health problems and new injury. A more nuanced picture emerged when injury occurrence was expressed relative to reconstructed accumulated training exposure rather than compared as raw proportions: the estimated incidence rate per 1,000 training hours was 8.98 in the course group and 10.56 in the team group (incidence rate ratio 1.18, 95% CI 0.79–1.75, *p* = 0.42). This interval is wide and compatible with the team group’s true per-hour rate being either somewhat lower or substantially higher than the course group’s, so this analysis does not provide clear evidence of a between-group difference in exposure-adjusted risk—but it likewise does not establish that the rates are truly equal. Group membership and training exposure were themselves strongly correlated (*r* = 0.88) and could not be cleanly separated in standard regression models—group retained a significant, though attenuated, association with injury even after adjustment for load, while load’s association attenuated substantially after adjustment for group. The large difference in cumulative injury burden between groups was substantially attenuated when injury occurrence was expressed relative to estimated training exposure. However, given the wide confidence interval, the reconstructed nature of the exposure measure, and the strong correlation between group membership and training exposure, the available data do not allow the independent contribution of training exposure to the observed group difference to be determined precisely. The most defensible summary of this study’s findings is therefore that university-team members experienced a substantially higher observed training exposure and unadjusted injury burden over 12 weeks, that this burden was smaller when expressed relative to estimated training exposure, and that this design cannot fully determine how much of the remaining difference arises from training volume itself, technical/competitive demands, selection into the team, prior injury history, or other unmeasured features of team membership.

Two findings are worth highlighting in more detail. First, after group membership was included in the model, the previous wave’s training load and recovery score did not show clear associations with substantial health problems at the subsequent assessment. These findings should be interpreted in light of the temporal structure of the study: training load and recovery represented the 7 days preceding one assessment, whereas the subsequent health-problem assessment occurred approximately 4 weeks later. The lagged analysis therefore does not directly test whether training load or recovery immediately preceding a health problem predicts short-term risk, and the null estimates should not be interpreted as evidence that training load or recovery are unrelated to health problems more generally. The group-only, load-only, and combined models (Wave-level health problem risk, above) showed that adding load to a group-only model changed the group estimate only modestly while the load estimate itself attenuated sharply. The strong correspondence between group membership and training exposure made it difficult to separate the independent associations of group context and training load in this dataset; we therefore do not interpret this as evidence that training load and recovery are causally unimportant—particularly given that training load itself was measured approximately (a single representative 7-day RPE rather than session-specific recordings), which would tend to attenuate its estimated association independently of collinearity with group, nor do we claim that load’s association with injury operates “through” group in a formal mediating sense, since no mediation analysis was conducted. This pattern is broadly consistent with evidence from other combat and martial arts disciplines that competitive or higher-exposure training status is associated with materially greater injury risk (5, 6) (see the exposure-adjusted analysis under Cumulative injury over 12 weeks, below). Consistent with the first two stages of the classical sequence-of-prevention framework for sports injury research (14), the present study describes the extent and distribution of the problem and explores associated factors; establishing the underlying causal mechanism and testing preventive interventions are appropriate next steps rather than claims this design can support. Second, prior 12-month injury remained associated with new injury during follow-up even after adjustment for group and baseline load, consistent with the general pattern in overuse-injury epidemiology that injury history is often one of the more robust individual-level risk markers, though—as with all associations in this study—this should be interpreted descriptively rather than causally. From a prevention standpoint, this finding supports pre-participation injury-history screening and targeted secondary-prevention measures for previously injured practitioners, consistent with general sports-injury-prevention frameworks (14).

The predominance of lower back, knee, and ankle injuries, and the higher proportion of overuse relative to acute injury types, is broadly consistent with the repetitive, high-amplitude jumping, landing, and spinal-flexibility demands characteristic of Wushu Taolu routines. Technical difficulty, repetitive landing load, and greater exposure to high-difficulty movements—all more prevalent in the university-team group (Table 1)—plausibly contribute to this site-specific pattern, although this study did not formally test these factors as predictors of injury site. This pattern is also consistent with prior descriptive surveys reporting the knee, ankle, and lower back as the most frequently affected sites in Wushu Taolu athletes (4) and moderate-to-high overall injury prevalence in wushu practitioners more generally (3). The fact that healthcare seeking was reported in only around three in five substantial-health-problem observations (59.3%; see Cumulative injury over 12 weeks, above) suggests possible under-treatment or under-reporting of lower-severity or self-managed problems, which merits further attention from a sports-medicine and public-health service-provision perspective. The absence of an independent association between the previous wave’s recovery score and subsequent health problems in the adjusted model should not be over-interpreted as evidence that sleep and recovery are unimportant in this population; shorter sleep has been linked to elevated injury risk in other athletic samples (7), and the present single-item, group-confounded analysis may simply have been underpowered to detect a within-person effect once group was included. The lower sleep and recovery observed in the team group may also reflect a bidirectional relationship between training and sleep rather than a simple unidirectional injury-risk pathway: in physically active adolescent and young-adult populations, greater exercise intensity has been associated with longer sleep duration, while sleep quality and duration have themselves been associated with subsequent physical activity intensity and duration (8, 9), so the higher training load and shorter sleep observed in the team group here may be mutually reinforcing rather than sleep being a simple downstream consequence of training volume. A further strength of the study is its prospective focus on university Wushu Taolu practitioners, a population that remains under-represented in the injury-surveillance literature, particularly outside Western team-sport settings.

## Limitations

Several limitations should be considered when interpreting these findings. First, and most importantly, the groups were not randomly assigned: course and team membership was determined by pre-existing enrollment and selection processes, and the two groups differed substantially at baseline in training experience, competition history, and prior injury. Group therefore acts as a proxy for a bundle of correlated exposures—training volume, technical difficulty, competition exposure, selection effects, and injury history among them—rather than a single, isolable factor, and the associations reported here should not be interpreted as evidence that team training per se causally increases injury risk. Second, and closely related, group membership and training load were highly collinear (*r* = 0.88 at baseline; variance inflation factors of approximately 5 for both variables in the multivariable models), which limits our ability to statistically separate their independent contributions; the group-only/load-only/combined model comparisons and the exposure-adjusted incidence-rate analysis (see Wave-level health problem risk and Cumulative injury over 12 weeks, above) were an attempt to partially address this, but they rely on an approximate reconstruction of training hours (weekly hours multiplied by an assumed 4-week duration per wave) rather than continuously monitored exposure time, and a more precise, prospectively logged measure of training-time exposure would allow a more definitive dose–response analysis. A sensitivity analysis reconstructing exposure from the mean of adjacent-wave training hours yielded a materially similar incidence-rate ratio (1.17 vs. 1.18), but neither approach can recover unobserved week-to-week variation in training volume. Third, this was a single-institution study of university Wushu Taolu practitioners aged 18–23 years, which limits external validity. The findings should therefore not be assumed to generalize directly to elite Wushu athletes, recreational practitioners, other age groups, students at other universities or training systems, or practitioners of other Wushu disciplines (e.g., sanda). In addition, the wave-based injury indicator could register at most one new-injury event per participant per ~4-week interval, so a participant experiencing more than one injury within the same interval was not fully captured by the cumulative-injury count (see Cumulative injury over 12 weeks, above). In addition, because the questionnaire did not explicitly distinguish recurrent injuries, exacerbations, or unresolved injuries from clinically distinct new events across successive waves, repeated reports—particularly those involving the same anatomical site—may not always represent independent injury episodes. Fourth, training load, sleep, recovery, and current health-problem status relied on self-report over a 7-day recall window, which is subject to recall and social-desirability bias; in particular, because weekly training load was reconstructed from a single representative 7-day RPE rather than session-specific RPE recordings, measurement error may be larger in a sport such as Wushu Taolu, where training demands can vary markedly between sessions. The new-injury and time-loss variables relied on a longer (approximately 4-week) recall window, which—despite the dedicated recall module used here to reduce under-ascertainment—may still be less precise than injury-surveillance methods with continuous or weekly contact, and the one-wave-lagged health-problem model (Wave-level health problem risk, above) spans a longer and less precisely defined interval than a strict “previous week predicts next week” design, so null findings for training load and recovery in that model should not be read as evidence that load and recovery are unrelated to injury risk on shorter timescales. The study-developed OSTRC-informed questionnaire used to assess health problems was not formally psychometrically validated or pilot-tested before use, which may have introduced measurement error and limits direct comparability with studies using the validated OSTRC instrument. Recovery was assessed using a single unanchored 0–10 self-report item, without verbal definitions of the scale endpoints, rather than a validated multi-item recovery instrument. The absence of verbal anchors may have led participants to interpret the scale endpoints differently, thereby reducing measurement precision and limiting the comparability of recovery scores with those reported in studies using anchored or validated recovery measures. Accordingly, findings involving recovery should be interpreted cautiously. Fifth, statistical power for the cumulative-injury model, while adequate by conventional events-per-variable heuristics (~15–16 events per predictor), does not support inclusion of a larger set of covariates or interaction terms; a Box–Tidwell test found no evidence against linearity in the logit for the continuous predictors in the primary cumulative-injury model, but this check was not performed for the wave-level GEE health-problem model, and multiple statistical tests were conducted across the three modeling stages without formal correction, so borderline findings (e.g., the Time × Group interaction for recovery, *p* = 0.079 for the T2 contrast) should be treated as hypothesis-generating. Finally, although retention was high (89.8%), four-wave completion was numerically lower in the course group than in the university-team group, and differential loss to follow-up cannot be completely excluded as a source of bias. The closely consistent available-case sensitivity analysis for the cumulative-injury outcome is reassuring, although it cannot eliminate the possibility of residual attrition bias. A direct comparison of completers and non-completers on injury-related characteristics (Sample and retention, above) likewise did not provide clear evidence of injury-driven informative dropout, though the small number of non-completers (*n* = 26) limited power to detect such an effect.

## Conclusion

Over a 12-week observation period, university-team Wushu Taolu practitioners showed substantially higher unadjusted training exposure and injury burden than course-based practitioners. However, this design cannot determine whether that difference arises from greater training volume itself, from technical or competitive demands, from selection effects, from prior injury history, or from other unmeasured features of team membership, since group and training exposure were too highly correlated to separate statistically. The exposure-adjusted estimate was substantially smaller than the crude cumulative-risk contrast, indicating that the large between-group difference in cumulative injury burden was markedly attenuated when injury occurrence was expressed relative to estimated training exposure. However, the available data do not allow the independent contribution of training exposure to the observed group difference to be determined precisely. Future research with continuously monitored, prospectively logged training-time data, ideally across multiple institutions, is needed to more precisely separate exposure volume from other group-level differences; in the interim, these findings support extending injury and health-problem surveillance to course-based as well as team-based Wushu Taolu practitioners, considering exposure (training-time) rather than group membership alone when prioritizing load-management and sports-medicine resources, and giving attention to prior injury history as a marker for targeted secondary prevention.

## Data Availability

The original contributions presented in the study are included in the article/Supplementary material, further inquiries can be directed to the corresponding author.
