# Supplementary material for: Training exposure, recovery, and injury incidence among course-based and competitive university Wushu Taolu practitioners: a 12-week prospective cohort study
Source: Front Public Health. 2026 Sep 17;14:1950026. doi: 10.3389/fpubh.2026.1950026 (PMC13626987; doi:10.3389/fpubh.2026.1950026)
Supplement: Supplementary file 1 [file Table_2.docx]

**Supplementary Material 1**

**Chinese-Language Health-Problem Questionnaire and English Reporting Translation**

*For: Training Exposure, Recovery, and Injury Incidence among Course-Based and Competitive University Wushu Taolu Practitioners: A 12-Week Prospective Cohort Study*

**Instrument-development note**

The health-problem questionnaire was developed in Chinese specifically for the present study and was informed by the conceptual structure of the Oslo Sports Trauma Research Centre (OSTRC) approach. It was not a direct translation or an item-by-item adaptation of the validated OSTRC questionnaire. No formal pilot testing, structured expert content-validation procedure, forward–back translation, or separate psychometric validation study was conducted before data collection. The English wording below is provided for reporting purposes only and was not administered to participants.

**Table S1. Health-problem items**

| **Item** | **Domain** | **Chinese-language item** | **English reporting translation** |
| --- | --- | --- | --- |
| C1 | Participation restriction | 过去7天，由于身体健康问题（如疼痛、不适或受伤），你参加武术套路训练或比赛的程度受到多大影响？ | During the past 7 days, to what extent did a health problem (such as pain, discomfort, or injury) affect your participation in Wushu Taolu training or competition? |
| C2 | Reduction in training volume | 过去7天，由于身体健康问题（如疼痛、不适或受伤），你的武术套路训练量减少了多少？ | During the past 7 days, to what extent was your Wushu Taolu training volume reduced because of a health problem (such as pain, discomfort, or injury)? |
| C3 | Impaired performance | 过去7天，由于身体健康问题（如疼痛、不适或受伤），你在武术套路训练或比赛中的表现受到多大影响？ | During the past 7 days, to what extent was your performance in Wushu Taolu training or competition affected by a health problem (such as pain, discomfort, or injury)? |
| C4 | Symptoms | 过去7天，你与武术套路训练或比赛相关的身体不适、疼痛或其他症状有多严重？ | During the past 7 days, how severe were any physical discomfort, pain, or other symptoms related to Wushu Taolu training or competition? |

**Table S2. Response categories and scoring**

| **Item** | **Score** | **Chinese response category** | **English reporting translation** |
| --- | --- | --- | --- |
| C1 | 0 | 完全参加训练或比赛，没有受到影响 | Full participation in training or competition without limitation |
| C1 | 8 | 能够参加训练或比赛，但有轻微影响 | Able to participate, but with a minor limitation |
| C1 | 17 | 参加训练或比赛的程度明显减少 | Participation in training or competition was moderately reduced |
| C1 | 25 | 完全无法参加训练或比赛 | Unable to participate in training or competition |
| C2 | 0 | 训练量没有减少 | No reduction in training volume |
| C2 | 8 | 训练量轻度减少 | Slight reduction in training volume |
| C2 | 17 | 训练量中度减少 | Moderate reduction in training volume |
| C2 | 25 | 训练量大幅减少 | Severe reduction in training volume |
| C3 | 0 | 训练或比赛表现没有受到影响 | No impairment in training or competition performance |
| C3 | 8 | 训练或比赛表现轻度受到影响 | Slight impairment in training or competition performance |
| C3 | 17 | 训练或比赛表现中度受到影响 | Moderate impairment in training or competition performance |
| C3 | 25 | 训练或比赛表现严重受到影响 | Severe impairment in training or competition performance |
| C4 | 0 | 没有症状 | No symptoms |
| C4 | 8 | 轻度症状 | Mild symptoms |
| C4 | 17 | 中度症状 | Moderate symptoms |
| C4 | 25 | 严重症状 | Severe symptoms |

**Scoring and definition of a substantial health problem**

• Each item was scored 0, 8, 17, or 25 according to the ordered response category selected.

• The four item scores were summed to produce a total health-problem severity score ranging from 0 to 100.

• A substantial health problem was defined as C2 ≥17, C3 ≥17, or C1 =25.

• C4 contributed to the total severity score but, by itself, was not used to define a substantial health problem.

**Interpretation note.** Because this was a study-developed, OSTRC-informed questionnaire rather than a formally validated Chinese version of the OSTRC instrument, its scores should not be regarded as psychometrically equivalent to scores obtained using the validated OSTRC questionnaire.

**Conceptual references**

1. Clarsen B, Myklebust G, Bahr R. Development and validation of a new method for the registration of overuse injuries in sports injury epidemiology: the Oslo Sports Trauma Research Centre (OSTRC) overuse injury questionnaire. Br J Sports Med. 2013;47:495–502. doi:10.1136/bjsports-2012-091524.

2. Clarsen B, Rønsen O, Myklebust G, Flørenes TW, Bahr R. The Oslo Sports Trauma Research Center questionnaire on health problems: a new approach to prospective monitoring of illness and injury in elite athletes. Br J Sports Med. 2014;48:754–760. doi:10.1136/bjsports-2012-092087.
